# Personality and psychopathology in potential live kidney donors: A cluster analysis of personality features

**DOI:** 10.1371/journal.pone.0221222

**Published:** 2019-08-14

**Authors:** César Leal-Costa, Antonio Jesús Ramos-Morcillo, Fermín Martínez Zaragoza, Purificación Bernabeu Juan, Jesús Rodríguez-Marín, María Ruzafa-Martínez, Carlos Javier van-der Hofstadt Román

**Affiliations:** 1 Nursing Department, Faculty of Nursing, University of Murcia, Murcia, Spain; 2 Instituto de Investigación Sanitaria y Biomédica de Alicante (ISABIAL—Fundación FISABIO), Alicante, Spain; 3 Health Psychology Department, Miguel Hernandez University (UMH), Elche, Spain; 4 Hospital Psychology Unit, University Hospital of Alicante, Alicante, Spain; Warszawski Uniwersytet Medyczny, POLAND

## Abstract

**Background:**

The previous psychosocial evaluation of the potential living kidney donors (PLKD) requires a detailed understanding of the psychosocial benefits and the possible damages of the act of donation.

**Objective:**

The aim was to create clusters by using the clinical patterns of personality and to evaluate their influence on psychopathological variables.

**Methods:**

Observational, analytical and cross-sectional study that included the PLKD from February 2009 to March 2017. The patients were referred to the Hospital Psychology Unit by the Transplant Coordination Unit. The total sample was composed of 100 participants. The socio-demographic characteristics, the relationship with the recipient and the Millon Clinical Multiaxial Inventory were included.

**Results:**

The final sample was composed by 100 PLKD. The mean age of the participants was 45.70, and most were women (70%). The analysis showed a final result of 3 personality clusters that best represented the data, in agreement with the DSM-5 classification. The PLKD from cluster 3 obtained greater scores in all the clinical syndromes.

**Conclusions:**

The personality evaluation of the PLKD could help with the planning of monitoring protocols of the participants who were classified to cluster 3, in order to improve their post-transplant psychosocial adjustment. This result makes us consider the usefulness of the psychosocial evaluation to preserve the psychological health of the PLKD.

## Introduction

The prevalence of chronic kidney disease has been progressively increasing each year worldwide, and it is now estimated to be between 10% and 16%, due to, at great length, to the increase in the number of diseases such as hypertension and diabetes [1]. A kidney transplant is the best therapeutic option for the majority of patients with end-stage renal disease (kidney failure), as it reduces morbidity and mortality, as compared with other kidney-replacement therapies [2,3].

There is currently a deficit between the individuals who require a kidney transplant and the kidneys available from deceased donors [4], so that a potential live kidney donor (PLKD) has become the possible solution for closing the gap between supply and demand. When the transplant comes from a live donor, the survival rate is greater, due to the higher quality of the organ, as well as the decreased wait time for the transplant [5].

The living donor candidates have been classified in different ways; thus Olbrisch et al. [6] divides them into six types (genetically related to the recipient, emotionally related with the recipient, altruists with a direct relationship with the recipient, altruists without a relationship with the recipient, organ sellers and people who participate in programs of cross donation, when a donation with the person with whom one has a relationship was not possible, either due to genetics or emotional reasons), and Fisher [7] divided them into two types, related donors (members of the biological family) and non-related (friends of the family, colleagues, church members, work colleagues, etc.).

Living kidney donation is a complex process that encompasses ethical, psychological, social and physical aspects. It is performed with the expectations that the risk for the donor will be compensated for by the psychosocial benefits (quality of life, social and psychological well-being, body image and concept of self) and the improvement of the recipient’s health. However, depression, anxiety and stress have also been described [8].

A detailed understanding of the psychosocial benefits and the possible damages caused by living kidney donation is fundamental for guiding the informed consent and promoting the maintenance of the psychological health of the donors, making the prior psychosocial evaluation of the candidate indispensable [7,9].

Most of the studies propose that the psychosocial evaluation encompass important areas, underlining mental health, the understanding of the donation risks, the analysis of the potential donor-recipient relationship, the motivation for donating, the strategies for coping with the surgery and the possible posterior complications, as important areas [6–10].

The evaluation of mental health should be performed to describe any premorbid psychiatric disease in the shape of any mental disorder (i.e. acute psychosis, schizophrenia, schizoaffective disorder), important state of mind disorders (depression, dysthymia), anxiety disorders (generalized anxiety, phobias, post-traumatic stress disorder, etc.) or personality disorders [7]. The final intent is to evaluate if there is a contraindication or limits to living kidney donation.

The psychosocial evaluation has been widely reviewed in the literature, and it has been shown that there is a great variety of protocols used for it. Therefore, validated tools have been used in some cases, while in others, semi-structured clinical interviews have been the norm. In a systematic review on the psychosocial health of the PLKD, [8] established that the most-utilized tools were general health, self-esteem, anxiety, stress, control locus, body image, quality of life and social support scales. Other studies have used the Alexithymia scales, the Mini Mental State Examination (MMSE), the Temperament and Character Inventory (TCI), the Multiphasic Minnesota Personality Inventory (MMPI) or the MMPI-2, the Millon Clinical Multiaxial Inventory-III (MCMI-III) and the Diagnostic and Statistical Manual-IV (DSM-IV) [11–15].

As stated in some studies, there seems to be an influence between the personality profiles of the PLKD and their psychological health. The dysfunctional personality profiles in the living donor can determine the coping strategies for the acting roles and independent decisions [14,16]. Thus, an evaluation of the personality before the donation, with a classification according to the personality cluster, would allow grouping the living donors based on their personality profiles, to be able to detect the more dysfunctional profiles and be able to monitor the individuals throughout the entire process of donation. However, there is still a gap in the identification of the characteristics of the donors before the process of donation that are related with a decreased post-donation psychological health [17].

For this reason, the general objective of the study was to create clusters by using the clinical patterns of personality and to evaluate their influence on the psychopathological variables of the live kidney donors.

## Method

### Design

Observational, analytical and cross-sectional study that included potential live kidney donors from February 2009 to March 2017 at the Unit of Hospital Psychology from the General University Hospital of Alicante, Spain.

### Participants

The sample was composed of participants who were accepted by the Transplant Unit for being evaluated as potential live kidney donors (PLKD). The inclusion criteria were: 1) that the donation was free, conscious and disinterested, 2) normal kidneys and 3) reduced risk of developing nephropathy in the long term. The exclusion criteria were: 1) having other diseases or alterations that could increase the risk during surgery or anesthesia, and 2) having diseases that could be transmitted to the recipient (cancer, infections, etc.).

### Measures

The instruments that were part of the evaluation protocol were:

Sociodemographic characteristics (age, gender, marital status, number of children and level of education) and relationship with the recipient (genetics, emotional and altruistic) which were collected with an “ad hoc” questionnaire used during the interview in the first consultation.

The Millon Clinical Multiaxial Inventory-III (MCMI-III) [18]. This is a benchmark instrument for the evaluation of personality and clinical disorders. This inventory is comprised of 175 items with a true-false dichotomous response format designed to be used with clinical populations. It contains 11 clinical personality pattern scales: schizoid, avoidant, depressive, dependent, histrionic, narcissistic, antisocial, sadistic (aggressive), compulsive, negativistic (passive-aggressive) and self-defeating; three severe personality pattern scales: schizotypal, borderline and paranoid; seven moderate clinical syndrome scales: anxiety disorder, somatoform disorder, bipolar-manic disorder, dysthymia, alcohol dependence, drug dependence and post-traumatic stress disorder; and three severe clinical syndrome scales: thought disorder, major depression and delusional disorder. The personality patters of the MCMI-III are related with Axis II, and the clinical syndromes with Axis I of the DSM-5 [19]. The MCMI-III correction is performed through the computerized application of the test. This application provides the direct scores and their conversion into prevalence scores (PREV). As for the interpretation, cut-off points were defined for the PREV scores. For the personality scales, the scores ranging from 75 to 84 informed about the presence of personality traits that were clinically significant, while scores 85 or higher suggested the presence of a disorder. For the clinical syndromes, the scores ranging from 75 to 84 indicated the presence of a disorder, and the scores 85 or higher denoted the prominence of a specific syndrome. The reliability evidence provided an internal consistency between 0.66 and 0.90, exceeding the value of 0.80 of the alpha coefficients in 20 scales. As for the test-retest reliability, the values found were 0.82 and 0.96 after 5–14 days. The external validity criteria showed correlations between the scores from the MCMI scales and the dimensions of the Minnesota Multiphasic Personality Inventory (MMPI), with the correlations being moderate-high (>0.5) for the related scales.

### Procedure

**Fig 1:**
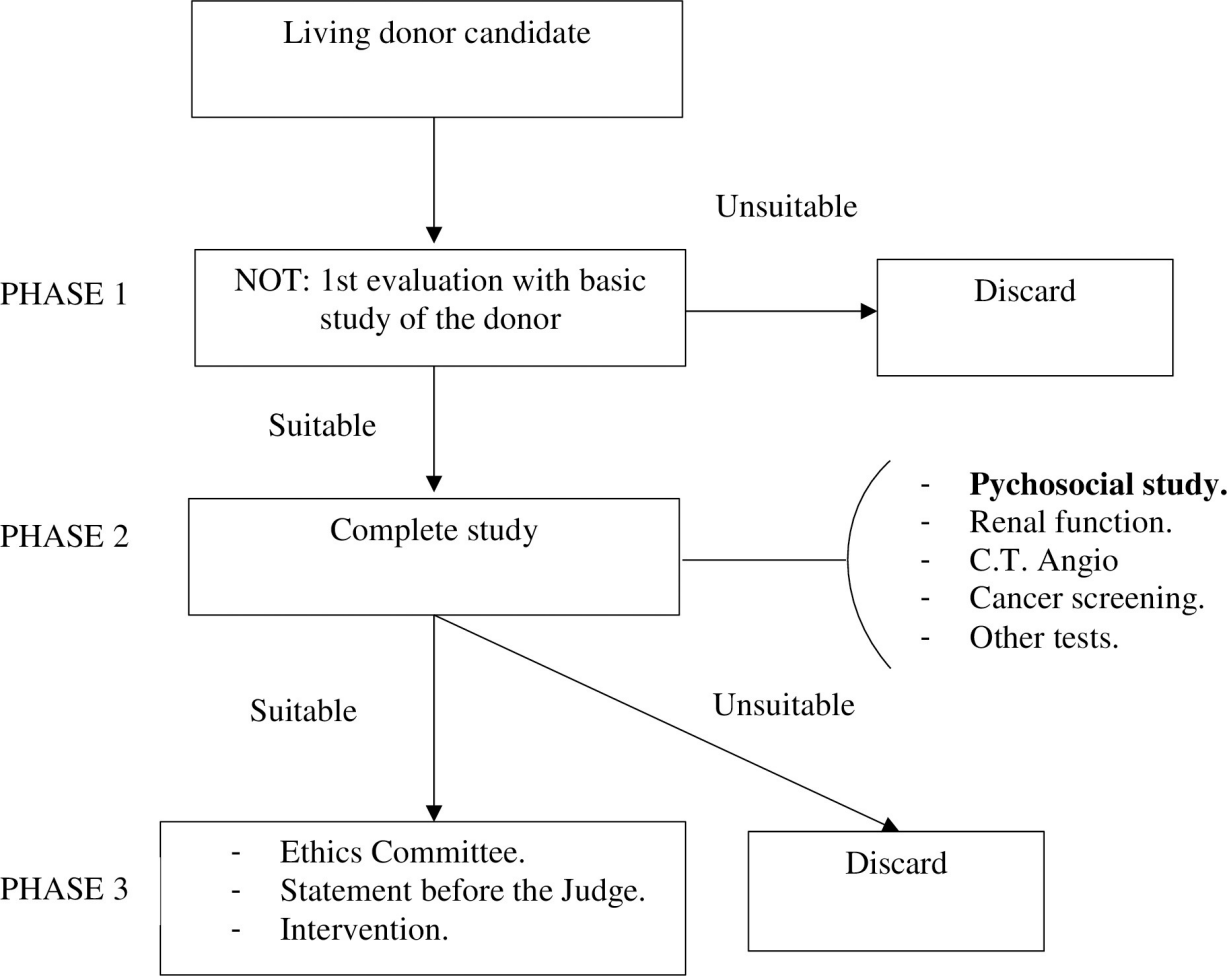
Flow chart of the phases of the procedure to be followed by a living kidney donor.

The patients went to the Unit of Hospital Psychology at the General University Hospital of Alicante, referred from the Transplant Coordination Unit. Once a potential donor was detected, 3 phases were established for performing the different evaluations (Fig 1). In phase 2 of the evaluation, the psychosocial study took place, conducted by psychologists from the Unit, and using the instruments described above. The first interview was conducted along with the application of the MCMI-II for the evaluation of the personality profiles, and a second one for the return of the results. The evaluations conducted were part of the evaluation protocol of the hospital psychology unit.

### Ethical considerations

The study protocol was checked and approved, in accordance with current law, by the Ethics Committee of Clinical Research at the General University Hospital of Alicante, with code PI2014/06, carried out with respect to the principles stated in the Declaration of Helsinki [20].

The participants received information about the study through an information sheet created about the research project, as well as an explanation provided by any of the project researchers. The participants who voluntarily accepted to participate in the study signed an informed consent form.

### Data analysis

In first place, a descriptive analysis was conducted. The statistics results used to describe the quantitative variables were the mean and standard deviation. In the case of the qualitative variables, the frequencies and percentages were used. With the aim of examining the personality profiles of the participants, a cluster analysis was conducted with the personality scales from the MCMI-III (axis II of the DSM-5) in order to identify homogeneous groups of patients with similar patterns. First, a hierarchal analysis was conducted using Ward’s method in order to estimate the optimum number of clusters by calculating the squared Euclidian distance between the measurement pairs. Afterwards, a non-hierarchal analysis was conducted to specify the number of clusters that we wanted to obtain (though K-means cluster analysis), allowing the statistical package to randomly select the centroids, so that the results could be validated. For the evaluation of which was the most-adequate solution (optimum number of clusters), two criteria were combined: a) the changes in the cluster coefficient when moving on to the next grouping stage, and b) the clusters established by the theory of severe personality profiles [19]. To analyze the correspondence of the clusters from the hierarchal and non-hierarchal analysis, a correlation analysis was conducted for the categorical values with Spearman’s Rho coefficient. Also, different analyses were performed in order to prove the validity of the clusters. First of all, a multivariate analysis of variance (MANOVA) was performed, using the clusters as the criterion and the scales of the clinical syndromes of the MCMI-III (axis I of the DSM-5) as the dependent variables. The null hypothesis stated that there was no effect from the clusters on the clinical syndromes of the MCMI-III. Afterwards, a series of analysis of variance (ANOVAs) were performed to find if there were differences between the clinical syndromes according to the clusters. The results were considered statistically significant at p<0.05. Before proceeding with the analysis, the assumption of normality was verified with the Kolmogorov-Smirnov test (p>0.05), and the homoscedasticity with Levene’s test (p>0.05). For the processing and analysis of data, the IBM SPSS statistical package V22.0 (New Castle, New York, USA) was used.

## Results

The final sample was composed by 100 PLKD. The participants represented the total potential kidney donors at the General Hospital of Alicante, Spain, during the data collection period. The mean age of the participants was 45.70 years (SD = 9.50), with most being women (70%), with a marital status of married or in a relationship (72%), and with primary (43%) and secondary (35%) education. Most (78%) had at least one child, and the mean number of children was 1.50 (SD = 1.05). As for the relationship with the recipient, 68% of them were genetically related, 27% had an emotional link, and 5% were altruistic donors without any relationship with the recipient. Lastly, among the individuals evaluated, 41 donated, 48 were unable to due to medical problems that were detected when the background study was concluded, 3 did not because the recipients had received the donation from a cadaver, and 8 were in-waiting (Table 1).

**Table 1:** Descriptive statistics of the sociodemographic characteristics, relationship with the recipient of the total and according to the cluster of the participants.

	Total n = 100	Cluster			p
		I n = 43	II n = 35	III n = 22	
Age M (SD	45.70 (9.50)	46.72 (8.77)	44 (9.60)	46.41 (10.72)	0.423^b^
Gender n (%)					
Men	30 (30)	10 (23.3)	8 (22.9)	12 (54.5)	0.017^a^
Women	70 (70)	33 (76.7)	27 (77.1)	10 (45.5)	
Marital status n (%)					
Single	15 (15)	5 (11.6)	6 (17.1)	4 (18.2)	0.519^a^
Married or in couple	74 (74)	34 (79.1)	23 (65.7)	17 (77.3)	
Separated or divorced	11 (11)	4 (9.3)	6 (17.1)	1 (4.5)	
Level of education n (%)					
Primary	44 (44)	15 (34.9)	16 (45.7)	13 (59.1)	0.333^a^
Secondary	35 (35)	16 (37.2)	14 (40)	5 (22.7)	
University	21 (21)	12 (27.9)	5 (14.3)	4 (18,2)	
Nº of children M (SD)	1.50 (1.05)	1.70 (1.08)	1.20 (0.93)	1.59 (1.10)	0.102^b^
Relationship with the recipient n (%)					
Genetics	68 (68)	31 (72.1)	24 (68.6)	13 (59.1)	0.737^a^
Emotional	27 (27)	11 (25.6)	9 (25.7)	7 (31.8)	
Altruistic	5 (5)	1 (2.3)	2 (5.7)	2 (9.1)	

^a^ χ^2^ test

^b^ *ANOVA-*test.

The score obtained on the 11 clinical patterns, the 3 severe personality pathologies, the 6 clinical syndromes and the 3 severe clinical syndromes of the MCMI-III, did not exceed a mean value higher than 75 (Table 2).

**Table 2:** Descriptive statistics of PREV scores for all MCM-III variables.

Total n = 100	M	SD	PREV>75 %	PREV>84 %
Personality patters scales				
Schizoid	37.94	19.61		
Avoidant	25.87	19.87	1	
Depressive	19.87	17.89	1	
Dependent	30.35	19.99	1	
Histrionic	63.83	15.54	26	5
Narcissistic	66.45	8.86	14	2
Antisocial	40.38	22.88	2	
Sadistic	37.58	22.52		
Compulsive	67.62	18.11	23	20
Negativistic	36.31	22.71	1	
Self-Defeating	18.55	18.61		
Schizotypal	19.02	20.53		
Borderline	21.35	20.42		
Paranoid	35.07	24.16	2	
Clinical Syndromes Scales				
Anxiety disorder	32.63	27.81	6	6
Somatoform disorder	20.62	20.48	1	
Bipolar-manic disorder	40.49	20.86	4	
Dysthymia	15.12	17.84		
Alcohol dependence	37.28	25.31		
Drug dependence	37.27	25.66	2	1
Post-traumatic stress disorder	18.04	19.81		
Thought disorder	18.89	18.76		
Major depression	14.73	16.87		
Delusional disorder	30.45	31.59	2	1

The percentages of the participants who obtained scores from 75 to 84 were calculated, with these scores suggesting the presence of personality traits that were clinically significant or the presence of a syndrome. The percentages of those who obtained scores of 85 or higher were calculated as well, suggesting the presence of a personality disorder and the prominence of a specific syndrome. With respect to the scales of the 11 clinical patterns and the 3 severe personality pathologies, the greatest percentage of participants who had some personality trait (scores from 75 to 84) were found in the scales “histrionic” (26%), “compulsive” (23%) and “narcissistic” (14%). On the other hand, the percentage of participants who had some personality disorder (scores 85 or higher) was lower, with the greatest percentages found in the scales “compulsive” (20%), “histrionic” (5%) and “narcissistic” (2%). For the clinical syndromes (scores from 75 to 84), the greatest percentage of participants who had some type of clinical syndrome was found in the scales “anxiety disorder” (6%), and “bipolar-manic disorder” (4%). On the other hand, the percentage of the participants who had a prominent clinical syndrome (scores 85 or higher) was less, with the greatest percentages found in the scale “anxiety disorder” (6%) (Table 2).

**Fig 2:**
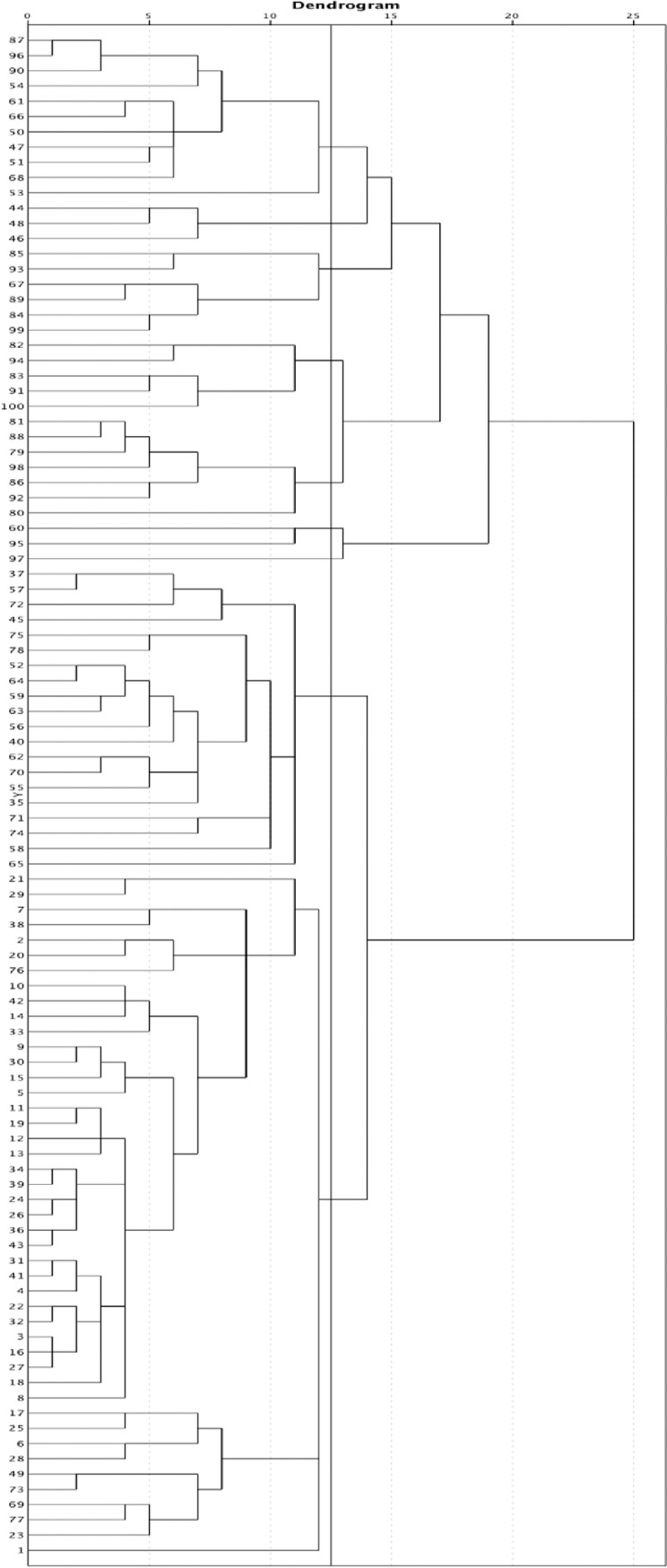
Dendrogram showing the levels of the hierarchical cluster.

The hierarchal and non-hierarchal analysis resulted in a final solution of 3 personality clusters that best represented the data. In the hierarchal analysis, to estimate the optimal number of clusters, the agglomeration coefficients were obtained, observing that the most important increase was found when moving from 3 to 2 clusters. On the other hand, the DSM-5 continues to classify the personality disorders into three groups based on their descriptive similarities. Table 3 shows the centers of the three clusters in the 14 personality scales of the MCMI-III, and Fig 2 shows the hierarchal dendogram with the sample of 100 participants. The non-hierarchal analysis of k-means was used to analyze the correspondence between the hierarchal and non-hierarchal analysis, with a correlation r_rho_ = 0.921 found between both solutions.

**Table 3:** Cluster centers for personality clinical scales of the MCMI-III.

	Cluster		
	1 n = 43	2 n = 35	3 n = 22
Schizoid	29	9	63
Avoidant	6	15	62
Depressive	6	5	45
Dependent	6	12	73
Histrionic	57	75	39
Narcissistic	69	71	73
Antisocial	6	81	17
Sadistic	6	72	60
Compulsive	99	16	93
Negativistic	6	69	63
Self-Defeating	6	40	48
Schizotypal	4	48	63
Borderline	4	68	51
Paranoid	4	60	79

**Fig 3:**
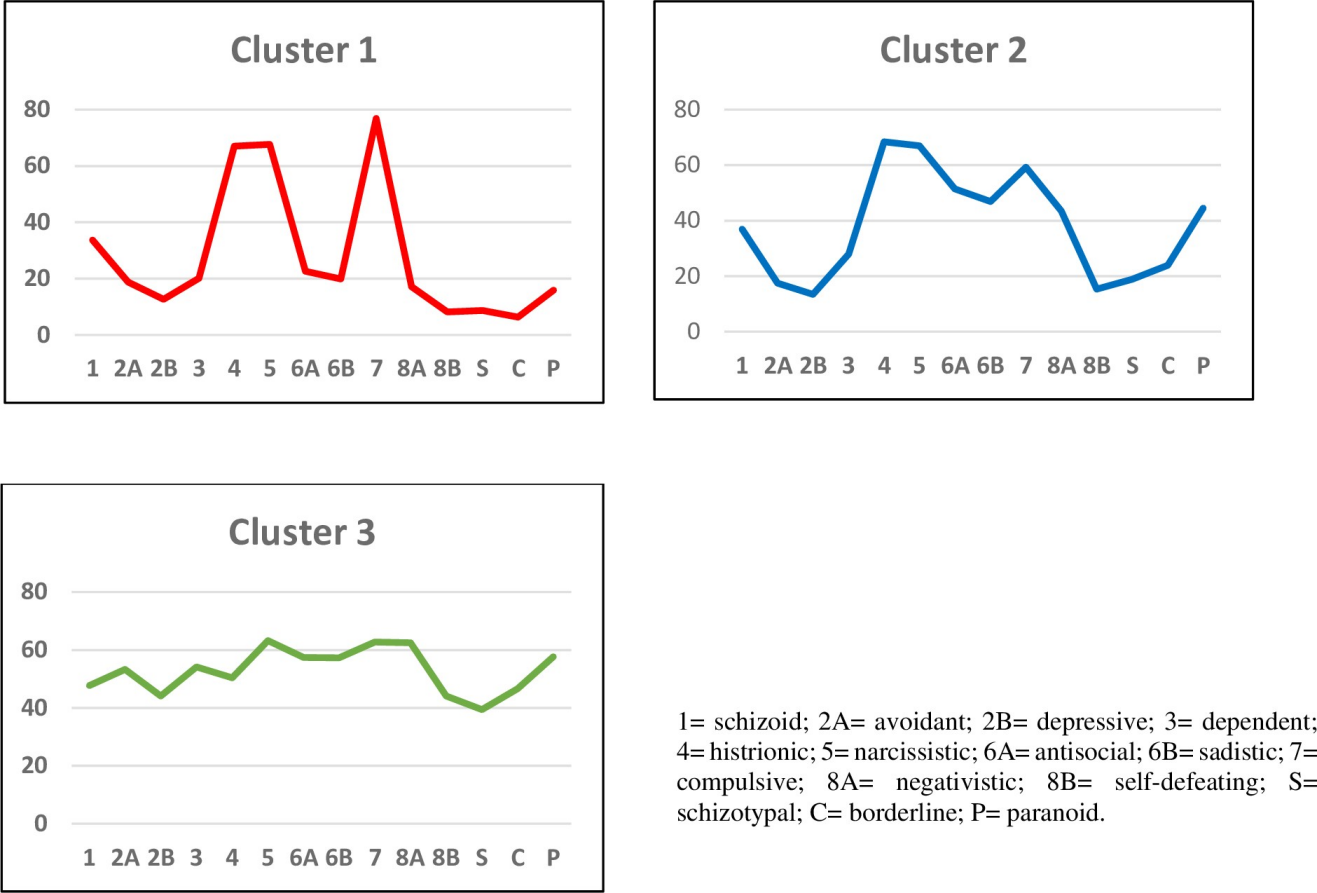
Mean scores of the sample in the personality clinical scales according to cluster.

The participants included in cluster 1 had low scores in all the personality scales except for the scales “histrionic”, “narcissistic” and “compulsive”, where they obtained higher scores as compared to the participants from the other two clusters. In cluster 1, the scale “compulsive” obtained a mean score > 75, and the scales “histrionic” and “compulsive” obtained similar scores. Also, it should be noted that the participants in this cluster obtained low scores in the scales “avoidant”, “self-defeating”, “schizotypal”, “borderline” and “paranoid”. The participants from cluster 2 obtained a higher score than the participants from cluster 1 and a lower one than the participants from cluster 3 in the personality scales of the MCMI-III, except for, (just as for cluster 1), the scales “histrionic”, “narcissistic” and “compulsive”, where they obtained a lower score as compared to cluster 1 but a greater one as compared to cluster 3. None of the scales from cluster 2 had a mean value higher than 75. In cluster 3, the participants obtained higher scores in all the personality scales of the MCMI-III, except for the scales “histrionic”, “narcissistic” and “compulsive”, where they obtained lower scores as compared to the other clusters. The participants included in this cluster obtained high scores in the scales “avoidant”, “dependent”, “sadistic”, “compulsive”, “negativistic” and “paranoid” (Fig 3).

**Fig 4:**
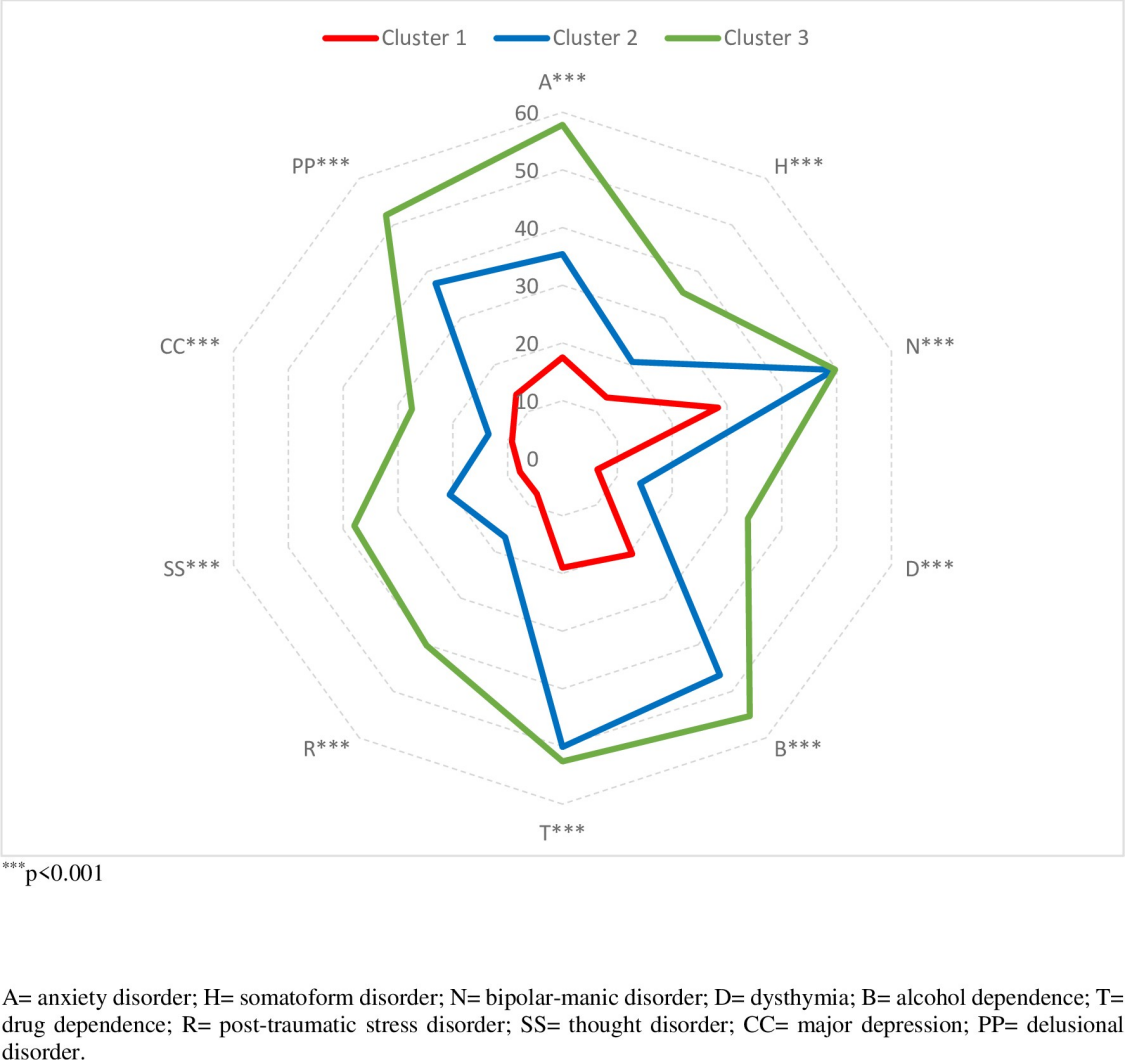
Comparison of means of clinical syndromes according to the different clusters.

In order to prove the validity of the cluster, a confirmatory multivariate analysis of variance (MANOVA) was performed using the three clusters as the criteria variable and the scales of the clinical syndromes as the dependent variables. The model was statistically significant (F_10,88_ = 109.31; p < .001; Eta^2^ = 0.925), so that the null hypothesis of the non-effect of the clusters on the clinical syndromes was rejected. Therefore, significant differences between the three clusters with respect to the set of variables exist. Next, a series of analysis of variance (ANOVA) were performed to verify if there were differences between the individual measurements, with statistically significant differences found according to the cluster among the clinical syndromes anxiety disorder (F_2,97_ = 22.26; p < .001; Eta^2^ = 0.315), somatoform disorder (F_2,97_ = 10.44; p < .001; Eta^2^ = 0.180), bipolar-manic disorder (F_2,97_ = 16.78; p < .001; Eta^2^ = 0.257), dysthymia disorder (F_2,97_ = 26.19; p < .001; Eta^2^ = 0.351), alcohol dependence (F_2,97_ = 25.90; p < .001; Eta^2^ = 0.348), drug dependence (F_2,97_ = 30.68; p < .001; Eta^2^ = 0.387), post-traumatic stress disorder (F_2,97_ = 32.03; p < .001; Eta^2^ = 0.398), thought disorder (F_2,97_ = 30.57; p < .001; Eta^2^ = 0.387), major depression (F_2,97_ = 10.26; p < .001; Eta^2^ = 0.175) and delusional disorder (F_2,97_ = 15.74; p < .001; Eta^2^ = 0.245). Fig 4 shows that the participants in cluster 3 obtained higher scores in all the clinical syndromes that the participants in clusters 1 and 2, and also that the participants in cluster 2 obtained higher scores than the participants from cluster 1 (S1 Table). After the psychosocial evaluation, two participants were excluded as possible donors due to having an active psychopathology that could affect their ability to decide about the donation. These two participants belonged to cluster 3.

## Discussion

In this study, a cluster analysis was performed with the clinical patterns of personality of the PLKD, in order to evaluate their influence on the associated psychopathology. This could help with detecting participants who are more vulnerable to the correct maintenance of their psychological health and who may have post-donation complications, which would justify the previous psychosocial evaluation of the candidate. As for the scores obtained in Axis I and Axis II, the average of score of the total sample did not exceed 75, with these results being similar to other studies that utilized the MCMI-III as an instrument for the evaluation of the PLKD [14,16]. The percentage of participants with scores 75–84, or above 85 were higher in the scales “histrionic”, “compulsive” and “narcissistic” from Axis II, and in the scales “anxiety disorder” and “bipolar-manic disorder” from Axis I.

Recent studies [14,16,21], have searched for the relationship between personality profiles and psychological factors of the PLKD, but none of them have conducted a multivariate analysis in order to classify the donors into homogeneous groups according to personality, analyzing their possible influence with the associated psychopathology.

In the cluster analysis with the personality scales of the MCMI-III (axis II of the DSM-5) for the identification of homogeneous groups of patients with similar patterns, three personality clusters were found that could be clinically interpretable and that generally coincided with the personality groups from the DSM-5 [19]. This was expected, as the groups of different personality disorders within the DSM-5 are based on similar symptoms.

The greatest percentage of personality disorders was found in cluster 3, followed by disorders from cluster 2. The predominance of disorders of cluster 3 has also been underlined in published studies on candidate patients for bariatric surgery [22,23].

According to the MCMI-III manual [18], the appearance of important increases in the histrionic, narcissistic and compulsive scales is frequent, mainly due to the absence of significantly high scores in the scales of severe pathology of personality and pathology from Axis I (DSM-5), reflecting strong personal qualities as well as moderate levels of self-esteem (narcissist) or sociability (histrionic). This is in line with the results shown in our study, where the participants found in clusters 1 and 2 scored high in these scales.

When analyzing the relationship of the personality clusters and the clinical syndrome of axis I, it was observed that the PLKD from cluster 3 obtained higher scores in all the clinical syndromes than the participants from clusters 1 and 2, with these differences being statistically significant and with an effect size between moderate and good in all the clinical syndromes. Also, the multivariate analysis of variance (MANOVA) verified that there were significant differences between the three clusters with respect to the clinical syndromes.

Two cases were found in the sample where the presence of an active psychopathology resulted in their rejection as live donors, which totaled 2% of the sample. This percentage was similar to that obtained in other studies [24].

When focusing on the sociodemographic characteristics and the relationship with the recipient according to personality cluster, it was noticeable that most of the participants were women, as opposed to the deceased donors, who were mostly men, according to the National Organization of Transplants in Spain in 2017 [25].

### Limitations

The limitations of the study that should be addressed includes the design of the cross-sectional study, which entailed that there was no evaluation after the donation that could help in demonstrating the importance of the possible posterior monitoring of the PLKD found in cluster 3. Another limitation was the sample selection, due to their recruitment from a single hospital. A multi-center study would help with having more samples and with improving the object of study.

Lastly, it would be convenient to have statistics data at the regional, national and international levels of all the living donors who have already been evaluated, as these data would help us calculate if our sample was representative at the regional level, and if it was comparable to the rates at the national and international level. At present, only statistical data of the kidney transplants conducted with living donors is available, which are inferior to the evaluations conducted, as not all the candidates donated for different reasons.

## Conclusions

It can be concluded that the PLKD classified into cluster 3 obtained higher scores in all the clinical syndromes from axis I of the DSM-5. An evaluation of the personalities of the PLKD could help us with the planning of a monitoring protocol of the participants classified into cluster 3, which could improve their post-transplant psychosocial adjustment, in order to prevent future psychopathological problems. This result leads us to consider the usefulness of the psychosocial evaluation that is performed with respect to the current legislation, in order to preserve the psychological health and the rights of the donors. Other studies have shown that psychological problems are risk factors for PLKDs, and that they may need post-donation psychological support [17].

## Supporting information

S1 Table Descriptive statistics of the clinical syndromes according to the cluster of the participants. (DOCX)
