# Supplementary material for: Personality and psychopathology in potential live kidney donors: A cluster analysis of personality features
Source: PLoS One. 2019 Aug 14;14(8):e0221222. doi: 10.1371/journal.pone.0221222 (PMC6693753; doi:10.1371/journal.pone.0221222)
Supplement: S1 Table — (DOCX) [file pone.0221222.s001.docx]

|  | **Cluster 1**  **n=43** | | **Cluster 2**  **n=35** | | **Cluster 3**  **n=22** | |
| --- | --- | --- | --- | --- | --- | --- |
|  | **M** | **SD** | **M** | **SD** | **M** | **SD** |
| **Clinical Syndromes Scales** |  |  |  |  |  |  |
| Anxiety disorder | 17.49^2,3^ | 17.99 | 35.40^1,3^ | 28.34 | 57,82^1,2^ | 23.47 |
| Somatoform disorder | 13.02^3^ | 16.42 | 20.06^3^ | 19.21 | 35.50^1,2^ | 22.14 |
| Bipolar-manic disorder | 28.37^2,3^ | 17.04 | 49.57^1^ | 19.66 | 49.73^1^ | 17.83 |
| Dysthymia | 6.33^3^ | 6.08 | 14.17^3^ | 14.62 | 33.82^1,2^ | 23.55 |
| Alcohol dependence | 20.58^2,3^ | 21.58 | 46.49^1^ | 22.05 | 55.27^1^ | 15.82 |
| Drug dependence | 19.00^2,3^ | 21.68 | 50.09^1^ | 19.47 | 52.59^1^ | 18.64 |
| Post-traumatic stress disorder | 7.63^2,3^ | 8.67 | 16.94^1,3^ | 18.42 | 40.14^1,2^ | 18.04 |
| Thought disorder | 7.74^2,3^ | 7.39 | 20.57^1,3^ | 19.08 | 38.00^1,2^ | 17.88 |
| Major depression | 9.23^3^ | 12.48 | 13.49^3^ | 14.29 | 27.45^1,2^ | 21.56 |
| Delusional disorder | 13.63^2,3^ | 23.23 | 37.51^1^ | 30.98 | 52.09^1^ | 30.32 |

S1Table. Descriptive statistics of the clinical syndromes according to the cluster of the participants.

The superscript number indicates the cluster with which it has statistically significant differences with p<0.05.
